# Proceedings of the EuBIC Winter School 2019

**DOI:** 10.1016/j.euprot.2019.07.002

**Published:** 2019-08-17

**Authors:** Dominik Kopczynski, Wout Bittremieux, David Bouyssié, Viktoria Dorfer, Marie Locard-Paulet, Bart Van Puyvelde, Veit Schwämmle, Alessio Soggiu, Sander Willems, Julian Uszkoreit

**Affiliations:** a Leibniz-Institut für Analytische Wissenschaften – ISAS – e.V., Bunsen-Kirchhoff-Str. 11, D-44139, Dortmund, Germany; b University of Antwerp, Antwerp, Belgium; c Institute of Pharmacology and Structural Biology, University of Toulouse, CNRS, UPS, Toulouse, France; d Bioinformatics Research Group, University of Applied Sciences Upper Austria, Hagenberg, Austria; e Novo Nordisk Foundation Center for Protein Research, University of Copenhagen. Denmark^1^; f Laboratory of Pharmaceutical Biotechnology, Ghent University, Ghent, Belgium; g Department of Biochemistry and Molecular Biology, University of Southern Denmark, Campusvej 55, 5230, Odense, Denmark; h Department of Veterinary Medicine, University of Milan, Milan, Italy; i Ruhr University Bochum, Faculty of Medicine, Medizinisches Proteom-Center, Gesundheitscampus 4, D-44801, Bochum, Germany

**Keywords:** EuBIC, Computational proteomics, Bioinformatics, Protein quantification, Date independent acquisition

## Abstract

The 2019 European Bioinformatics Community (EuBIC) Winter School was held from January 15th to January 18th 2019 in Zakopane, Poland. This year’s meeting was the third of its kind and gathered international researchers in the field of (computational) proteomics to discuss (mainly) challenges in proteomics quantification and data independent acquisition (DIA). Here, we present an overview of the scientific program of the 2019 EuBIC Winter School. Furthermore, we can already give a small outlook to the upcoming EuBIC 2020 Developer’s Meeting.

## 1 Introduction

**Fig. 1:**
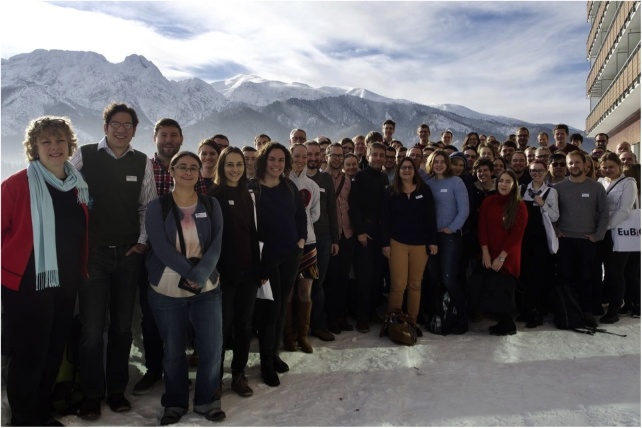
Participants of the EuBIC Winter School 2019. In the background, the mountains surrounding the conference venue in the city of Zakopane can be seen.

The 2019 EuBIC Winter School on proteomics bioinformatics, held from January 15th to January 18th 2019 in Zakopane, Poland, was the third conference of its kind organised by the European Bioinformatics Community (EuBIC, [1,2]), an initiative of the European Proteomics Association (EuPA) for user-oriented bioinformatics. The Winter School brought together scientists from both academia and industry in the field of computational mass spectrometry (MS)-based proteomics to present and discuss their research in workshops, keynote lectures, flash talks and poster presentations. The 93 participants (see Fig. 1) came from 18 different European countries, together with guests from the USA, Canada and China. Besides the scientific programme, which included 12 workshops, 10 keynote talks, 10 flash talks and 35 poster presentations, the participants were also given time to network, socialize and meet representatives of our main sponsors BSi, ProFI, Thermo Fisher Scientific and SVA. To trigger interactions and alleviate the mood, the conference bags contained tongue-in-cheek advice, such as “cite the tools you use”, “do calculate FDRs” or “join EuBIC” as well as a personal “Conference Bingo” sheet. For the Bingo, each participant had a number of words and events to cross out, which might occur in a talk (“open data”, “I would like to thank…”, “somebody starts clapping too early”, “presentation with font too small to read”). This Bingo culminated on the final day with a “BINGO” shout mid- session as a participant completed his version by crossing off “Speaker offers post-doc position”. Finally, as the main social event of the Winter School, a pub quiz with questions concerning proteomics and informatics with free drinks was hosted by the organizers at a local venue in Zakopane.

As the EuBIC has a special focus on teaching and education, the first day of the Winter School was dedicated to educational workshops on computational MS-based proteomics organised primarily by the community itself. On the second and third days the invited speakers and sponsors gave interactive keynotes in the morning and more advanced workshops in the afternoon. The fourth and final day consisted of another half day of keynotes given by the invited speakers and final discussions. The full conference program can be consulted online.^2^

## 2 Workshops

The first day of the Winter School featured two full day workshops and two half-day workshops on four different educational topics in parallel: (1) introduction to computational mass spectrometry using OpenMS (H. Röst, L. Bichmann and O. Alka) (2), computational introduction into data independent acquisition (DIA) (M. Dhaenens and B. Searle), and (3a) Label-free quantification: concepts and algorithms and (3b) quantitative proteomics: statistics, clustering and complexes (D. Bouyssié and V. Schwämmle). These workshops consisted partly of presentations giving an overview of the specific topic as well as hands-on tutorials to encourage the participants to work with these concepts.

After the keynote lectures on the second day, the participants could attend one of four parallel workshops organised by the invited speakers and sponsors. M. Eisenacher and J. Uszkoreit talked about “The essentials before and after spectrum identification: Selecting the appropriate database and inference strategy” [3] and guided participants through practicals exploring the protein database exports of the UniProt KB [4], analyses in the workflow environment KNIME [5] and performing protein inference using PIA [6,7]. In the workshop entitled” Quality Control and Benchmarking of Label-Free Quantification Workflows with LFQBench” S. Tenzer and M. Lacki explained how to apply the R package LFQBench [8] to proteomics data and how to interpret the results. Furthermore there were two workshops of our main sponsors: “Discovering the open-source Proline software suite, a new efficient and user friendly solution for label-free quantification” by ProFI (http://www.profiproteomics.fr/) and a workshop organised by ThermoFisher Scientific about the new features of Proteome Discoverer 2.3.

On the afternoon of the third day, four more workshops were offered. L. J. Jensen presented the “Network visualization with Cytoscape and stringApp” [9] with hands-on practicals, while M. Wilhelm presented a “Validation of peptide identifications” using ProteomicsDB [10]. In parallel,

E. Petsalaki guided the participants through an analysis using SELPHI in “SELPHI: using data- driven approaches for analysis of phosphoproteomics datasets” [11] in the first half of the workshop before F. Meier who showed the “Advanced data acquisition methods with MaxQuant.Live” [12] during the second half.

## 3 Keynotes

For this year’s Winter School we had in total ten keynote slots with 45 min talk and discussion each: four on both Wednesday and Thursday and two on Friday before the closing session. In keeping with the tradition of the first Winter Schools, the presenters were asked to talk about problems and challenges in the field of bioinformatics for proteomics they encountered during their recent research. To emphasize this, this year’s special focus was “Challenges in Quantification and DIA”.

On Tuesday, after the welcome and announcement, B. Searle (Institute for Systems Biology, Seattle, WA, USA) kicked off the keynotes with a talk about a new database search engine to detect genetic variants from DIA data using the tool XCorDIA [13]. After this, H. Röst (The Donnelly Centre for Cellular and Biomolecular Research, University of Toronto, Toronto, Canada) spoke about developing tools for the personalized medicine revolution and using mass spectrometry for longitudinal molecular profiling [[14], [15], [16]]. Here, he showed how new tools could be used to align the features of mass spectrometry analyses measured over a long time period using spanning trees. S. Tenzer (Institute for Immunology, University Medical Center of the Johannes-Gutenberg University Mainz, Mainz, Germany) highlighted how his lab was able to perform label-free quantification of complex proteomes using the still emerging ion-mobility-based DIA approaches [17]. Additionally, he noted the value and importance of reproducible chromatographic approaches and recent advances of microflow methods in this area. The fourth talk on this day was given by M. Eisenacher (Ruhr University Bochum, Bochum, Germany). He talked about historical researches which later were falsified, but seemed to be a relevant hypothesis at their time. With respect to this, he stated the question about the existence of a “reverse translatase” and its hypothetical applications in proteomics.

On the third day, the first talk was given by L. J. Jensen (University of Copenhagen, Copenhagen, Denmark) about STRING [18] and recent updates to its database. Exemplary stated in this regard were the text mining procedures and performances used to find protein-protein-interactions. M. Wilhelm (Chair of Proteomics and Bioanalytics, Technical University of Munich, Freising, Germany) presented Prosit, a tool to predict MS/MS spectra from a given peptide sequence, which was trained on the data generated by the ProteomeTools project [19,20]. The following talks on Thursday were oriented towards MS-based data analysis and how to drive biological insights from the huge amount of data generated nowadays. E. Petsalaki (EMBL-EBI, Wellcome Genome Campus, Hinxton, UK) discussed re-analysing phosphoproteomics data (“Using phosphoproteomics data to study context-specific signalling”) and illustrated the associated challenges [21]. Then, K. Lilley (University of Cambridge, Cambridge, UK) ended the morning session with a talk entitled “Insights into the multi-functioning proteome” where she exchanged views on the importance and difficulties of accurately measuring the proteome system-wise in a space- and time-defined fashion [22].

On the last day, S. Degroeve (VIB-UGent Center for Medical Biotechnology, Ghent, Belgium) presented Ionbot, a fully data-driven search engine for open modification and mutation searches. He highlighted the importance of accurate MS/MS spectra prediction [[23], [24], [25]] and could directly connect his thoughts to the talk of M. Wilhelm the day before. The final keynote of the Winter School was about trapped ion mobility spectrometry, which adds a new dimension for mass spectrometry-based proteomics and was given by F. Meier (Proteomics and Signal Transduction, Max Planck Institute of Biochemistry, Martinsried, Germany) [26].

## 4 Flash talks and poster presentations

**Fig. 2:**
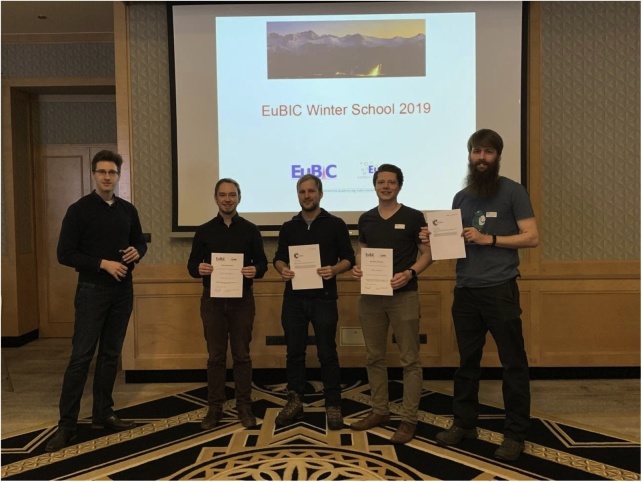
The winners of the flash talks and poster presentations. From left to right: Dominik Kopczynski of the organizing committee, the poster price winners Tim van den Bossche and Leon Bichmann, followed by the winners of the flash talks Ralf Gabriels and Henning Schiebenhöfer.

Each participant was encouraged to present their recent research in a poster presentation. Ten of the 35 submitted and peer reviewed posters were selected for a five-minute flash talk of the presented topic. The keynote speakers had the responsibility to vote for the best flash talk and the best poster. This was undoubtedly hard work during the lively poster session, which started directly after the workshops and lasted until well after midnight. During this time, all participants had the opportunity to talk about their research and discuss ideas about bioinformatic analyses of proteomics data. The awards for the best flash talk were given to Ralf Gabriels (VIB-UGent Center for Medical Biotechnology, Ghent, Belgium) and his talk on “MS2 peak intensity prediction (MS2PIP) for specific PTMs, fragmentation techniques and instruments” and Henning Schiebenhöfer (Robert Koch-Institute, Berlin, Germany) with “Prophane – Metaproteomic Data Analysis and Interpretation Made Simple”. The awards for the best poster went to Tim van den Bossche (VIB-UGent Center of Medical Biotechnology, Ghent, Belgium) and the poster “Prediction-based reduction of the search space in metaproteomics” and Leon Bichmann (University of Tübingen, Tübingen, Germany) for the poster “Immunopeptidomics of human tissues using DDA and DIA” (Fig. 2).

## 5 Outlook

In this third EuBIC Winter School, we were again able to attract many renowned keynote speakers presenting their interesting and exciting research topics in proteomics. Even if the number of 93 participants was slightly smaller than the previous edition, we are still very happy about the success of the conference. A post-conference survey confirmed the organizers’ impression of the participants’ general high satisfaction. The few negative experiences included the rather expensive venue, but most attendees will very likely join another EuBIC event and about 20% are even eager to have a longer Winter School with more workshops next time.

Next year’s EuBIC conference will again be a *Developer’s Meeting*, like the one in 2018 [2], with a more *hackathon*-like program comprising more hands-on coding and less keynote talks. The meeting will take place in Denmark in January 2020 and the planning of the program is already underway.

We, the organizing committee, emphasize that any member of the community who wants to become active in the EuBIC can join our channels^3^ and help us in ongoing and future activities with as much effort as each one has available. We would also like to remind everyone in the proteomics community that we have a special “Q&A” mailing list^4^ for all bioinformatics for proteomics or mass spectrometry related questions that often yields prompt answers by the community.

## Declaration of Competing Interest

The authors declare no conflict of interest.

## Footnotes

2 https://github.com/eubic/eubic_winterschool_programme/releases/tag/final2019.

3 https://www.proteomics-academy.org/bioinformatics

4 https://qa.proteomics-academy.org/
